# Metasurface-assisted orbital angular momentum carrying Bessel-Gaussian Laser: proposal and simulation

**DOI:** 10.1038/s41598-018-26361-0

**Published:** 2018-05-23

**Authors:** Nan Zhou, Jian Wang

**Affiliations:** Wuhan National Laboratory for Optoelectronics, School of Optical and Electronic Information, Huazhong University of Science and Technology, Wuhan, 430074 Hubei China

## Abstract

Bessel-Gaussian beams have distinct properties of suppressed diffraction divergence and self-reconstruction. In this paper, we propose and simulate metasurface-assisted orbital angular momentum (OAM) carrying Bessel-Gaussian laser. The laser can be regarded as a Fabry-Perot cavity formed by one partially transparent output plane mirror and the other metasurface-based reflector mirror. The gain medium of Nd:YVO_4_ enables the lasing wavelength at 1064 nm with a 808 nm laser serving as the pump. The sub-wavelength structure of metasurface facilitates flexible spatial light manipulation. The compact metasurface-based reflector provides combined phase functions of an axicon and a spherical mirror. By appropriately selecting the size of output mirror and inserting mode-selection element in the laser cavity, different orders of OAM-carrying Bessel-Gaussian lasing modes are achievable. The lasing Bessel-Gaussian_0_, Bessel-Gaussian_01_^+^, Bessel-Gaussian_02_^+^ and Bessel-Gaussian_03_^+^ modes have high fidelities of ~0.889, ~0.889, ~0.881 and ~0.879, respectively. The metasurface fabrication tolerance and the dependence of threshold power and output lasing power on the length of gain medium, beam radius of pump and transmittance of output mirror are also discussed. The obtained results show successful implementation of metasurface-assisted OAM-carrying Bessel-Gaussian laser with favorable performance. The metasurface-assisted OAM-carrying Bessel-Gaussian laser may find wide OAM-enabled communication and non-communication applications.

## Introduction

Orbital angular momentum (OAM) describing the “phase twist” (helical phase front) of light beams^1^, has recently gained increasing interest due to its potential applications in many diverse areas ranging from optical manipulation to optical communications^2–9^. Particularly promising is the use of OAM in free-space, fiber-based and underwater optical communications^10–16^. Since coaxially propagating OAM beams with different azimuthal OAM states are mutually orthogonal, it holds the potential to tremendously increase the capacity of communication systems similar to the wavelength-division multiplexing (WDM) technique. For various OAM-enabled applications the generation of OAM-carrying light beams is of great importance. Actually, the well-known high-order Bessel beams carry OAM. Moreover, Bessel beams have the distinct properties of suppressed diffraction divergence and self-reconstruction during propagation through uniform media^17,18^. All of these features have caught extensive attention. Owing to these attractive properties, Bessel beams have been widely applied in many fields such as microfabrication, optical trapping, imaging, laser scanning microscopy and optical communications^19–27^. However, considering the infinite transverse electrical field distribution and energy of Bessel beams, it is practically impossible to produce the perfect Bessel beams. In order to solve this problem, Bessel-Gaussian beams are often referred to replace perfect Bessel beams in practical applications. Bessel-Gaussian beams are able to contain finite energy and conserve approximate electrical field profiles of perfect ones over the distance defined by their waist radius. To generate Bessel-Gaussian beams effectively, one can illuminate the axicon with corresponding Laguerre-Gaussian beams. Various techniques for generating Bessel-Gaussian beams based on axicon have been put forward over the past years^28–30^. However, the methods of Bessel-Gaussian beam generation from axicons always suffer from axial nonuniformity and fabrication challenge^31^. As a substitution of axicon, metasurface, a two-dimensional equivalent of metamaterials, can provide an alternative by scattering the light beams using the thin array of sub-wavelength structured resonators^32–34^. Compared to conventional focusing elements such as optical lens and planar focusing devices demonstrated at optical frequencies^35–39^, metasurface has compact structure and flexible design that provide the capability to tailor the phase of the transmitted light from 0 to 2π to realize full control of optical wavefront. Metasurface facilitates volume reduction of optical elements and flexible spatial structure manipulation of light beams. Most of the metasurfaces can be divided into two categories, i.e. plasmonic metasurfaces based on plasmon resonances^40–42^ and dielectric metasurfaces based on electric/magnetic dipole (Mie resonances)^43,44^. Dielectric metasurface benefits the alleviation of loss compared to plasmonic metasurface. Beyond all-dielectric elliptical metasurface array^44^, hybrid dielectric/metal metasurface structure adding metal at the bottom as the reflection layer may further improve the efficiency and bring superior performance. In addition to the passive method of Bessel-Gaussian beam generation through axicon-enabled light beam conversion, an active method of Bessel-Gaussian beam laser is of great interest^45–49^. A digital laser with an intra-cavity digitally addressed holographic mirror was proposed and demonstrated with impressive performance for on-demand laser modes, i.e. customizing the output beam shape^49^. Additionally, in order to meet the requirement of compact Bessel-Gaussian beam laser, metasurface-based reflector can be employed to replace relatively complicated volume structure components. In this scenario, one would expect to see a metasurface-assisted OAM-carrying Bessel-Gaussian laser.

In this paper, a Bessel-Gaussian laser structure using metasurface as the resonator reflector is designed. The gain material of Nd:YVO_4_ enables the lasing wavelength at 1064 nm. Taking advantages of Fox-Li algorithm^50^, the generation of Bessel-Gaussian modes carrying different OAM values and the laser properties are comprehensively analyzed. The metasurface fabrication tolerance and the laser performance dependence are also discussed. The simulation results show favorable performance of the presented metasurface-assisted OAM-carrying Bessel-Gaussian laser.

## Results

### Concept and principle

**Figure 1:**
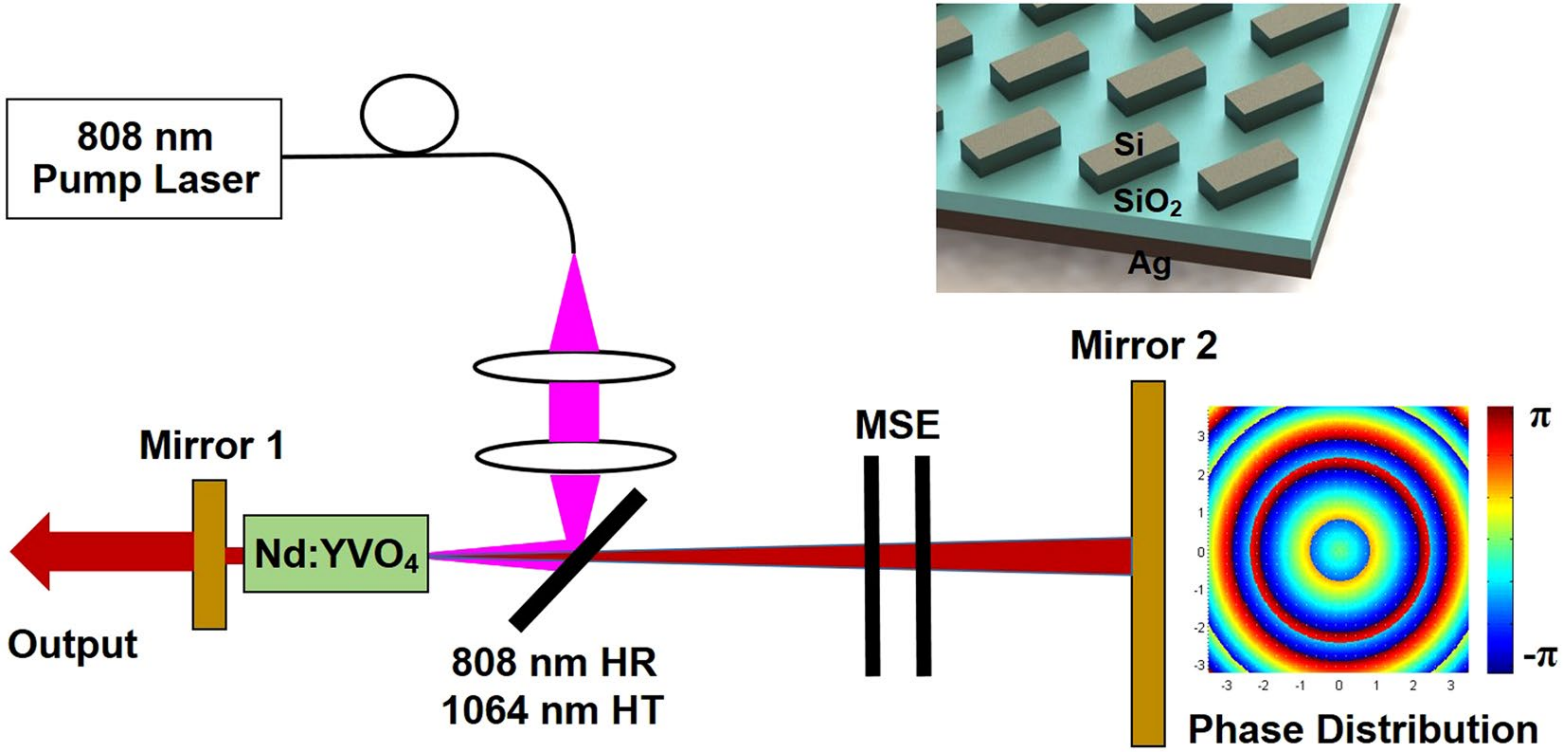
Schematic structure of the proposed Bessel-Gaussian laser with Nd:YVO_4_ as gain material, 808 nm diode laser as pump, two mirrors forming a Fabry-Perot (FP) cavity, and mode-selection element (MSE). Mirror 1: partially transparent plane mirror. Mirror 2: a combined structure of an axicon and a spherical mirror (e.g. concave mirror), which can be replaced by a metasurface structure.

The schematic structure of the proposed Bessel-Gaussian laser is illustrated in Fig. 1. The laser can be regarded as a Fabry-Perot (FP) cavity formed by two mirrors (mirror 1, mirror 2). In order to realize a Bessel-Gaussian laser, mirror 1 can be a partially transparent plane mirror, while mirror 2 is usually a combined structure of an axicon and a spherical mirror (e.g. concave mirror)^51^. The combined functions of an axicon and a spherical mirror can be achieved by a metasurface (sub-wavelength structured plane mirror) benefiting from its flexible spatial light manipulation. Hence, here mirror 2 is replaced by a metasurface reflector to construct the resonator configuration. Nd:YVO_4_ provides the cavity gain. A 808 nm laser serves as the pump. The top inset of Fig. 1 displays the schematic diagram of the designed dielectric metasurface (rectangular meta-reflection array), which is structured silicon (Si) antenna array on silica (SiO_2_) with an Ag bottom layer enhancing the efficiency. The right inset of Fig. 1 depicts the desired typical phase distribution (combined functions of an axicon and a spherical mirror) of mirror 2, which will be replaced by the metasurface structure in the designed metasurface-assisted OAM-carrying Bessel-Gaussian laser.

Besides the metasurface-based reflector, the mode-selection element (MSE)^48^ is necessary to be placed in the resonator for direct generation of Bessel-Gaussian modes with controllable handedness of the helical phase. In general, Bessel-Gaussian modes with opposite topological charges (right-handed and left-handed helical phase trajectories) have the same spatial intensity distribution. That is, except for the zero-order mode, the Bessel-Gaussian modes with opposite topological charges always coexist in a laser resonator, leading to petal-shaped intensity distributions^51–54^. The principle of MSE is to break such degeneracy by exploiting the fact that standing-wave intensity distributions for the Bessel-Gaussian modes with opposite topological charges inside the laser resonator are different^48^. Inside the laser resonator, both Bessel-Gaussian_01_^+^ or Bessel-Gaussian_01_^−^ modes have a two-lobe standing-wave intensity distribution rotating in opposite directions by 2π for every wavelength propagation. By appropriately putting structures with nanoscale thickness, a differential loss can be introduced between Bessel-Gaussian_01_^+^ and Bessel-Gaussian_01_^−^ modes, facilitating selective lasing of Bessel-Gaussian_01_^+^ or Bessel-Gaussian_01_^−^ mode.

**Figure 2:**
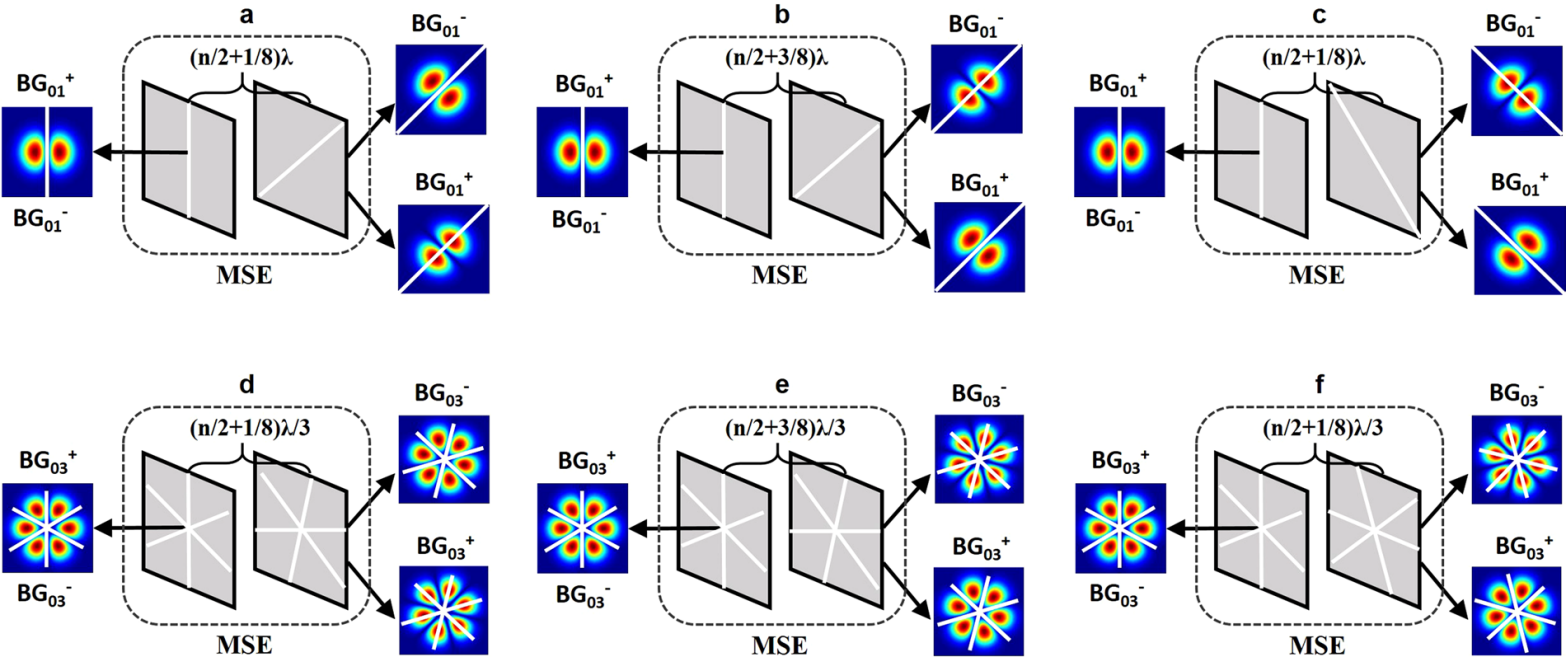
Operation principle of selective lasing of Bessel-Gaussian modes using mode-selection element (MSE). (**a**) Bessel-Gaussian_01_^+^ suppression and Bessel-Gaussian_01_^−^ lasing. (**b**,**c**) Bessel-Gaussian_01_^+^ lasing and Bessel-Gaussian_01_^−^ suppression with different sets of angle and distance between two nanoscale thickness wires (white lines). (**d**) Bessel-Gaussian_03_^+^ suppression and Bessel-Gaussian_03_^−^ lasing. (**e**,**f**) Bessel-Gaussian_03_^+^ lasing and Bessel-Gaussian_03_^−^ suppression with different sets of angle and distance between two nanoscale thickness wires (white lines). BG: Bessel-Gaussian.

The detailed operation principle of the MSE can be understood in Fig. 2. For selective lasing of Bessel-Gaussian_01_^+^ or Bessel-Gaussian_01_^−^ mode, as illustrated in Fig. 2a, two nanoscale thickness wires (white line) with an angle of 45° are put at the center of the mode. The first wire determines the available standing-wave intensity distribution of Bessel-Gaussian_01_^+^ and Bessel-Gaussian_01_^−^ modes at the position of the wire in the laser cavity. The initial condition is set by the first wire. The two-lobe standing-wave intensity distribution of Bessel-Gaussian_01_^+^ and Bessel-Gaussian_01_^−^ modes rotates anti-clockwise by an angle of −(n + 1/4)π and clockwise by an angle of (n + 1/4)π after propagation of (n/2 + 1/8)λ, where the second wire (45° with respect to the first wire) is positioned. It is noted that the second wire has almost no touch with Bessel-Gaussian_01_^−^ mode but full contact with Bessel-Gaussian_01_^+^ mode, resulting in minimum and maximum losses for Bessel-Gaussian_01_^+^ and Bessel-Gaussian_01_^−^ modes, respectively. Hence, the lasing of the Bessel-Gaussian_01_^+^ mode with relatively large induced loss by the second wire is suppressed. The opposite situation is achievable (Bessel-Gaussian_01_^+^ lasing and Bessel-Gaussian_01_^−^ suppression) simply by changing the distance between the two wires to be (n/2 + 3/8)λ, as shown in Fig. 2b. Alternatively, one can also obtain the opposite situation by keeping the distance between the two wires to be (n/2 + 1/8)λ while rotating the second wire with an angle of −45° with respect to the first wire, as shown in Fig. 2c. Remarkably, selective lasing of Bessel-Gaussian_01_^+^ or Bessel-Gaussian_01_^−^ mode is also achievable using a different group of angle and distance between the wires. Similarly, selective lasing of Bessel-Gaussian_03_^+^ or Bessel-Gaussian_03_^−^ mode can be realized assisted by MSE with proper angle and distance for the two sets of nanoscale thickness wires, as shown in Fig. 2d,e. Different from the situation for Bessel-Gaussian_01_^+^ and Bessel-Gaussian_01_^−^ modes, here in each position multiple wires are employed instead of a single wire, which is determined by the number of lobes of the standing-wave intensity distribution of high-order Bessel-Gaussian modes (e.g. three crossed wires for six-lobe standing-wave intensity distribution of Bessel-Gaussian_03_^+^ and Bessel-Gaussian_03_^−^ modes).

Remarkably, the output beam of a laser is determined by resonance geometric parameters such as resonance cavity length, radius curvature and dimensions of reflector. To describe Bessel–Gaussian modes of the designed laser resonator configuration, the transverse electrical field of a Bessel-Gaussian mode at a distance *z* from the waist in the paraxial approximation can be expressed as^51^

1 $${A}_{m}(\rho ,\phi ,z)=a\frac{{w}_{0}}{w(z)}{e}^{i{\rm{\Phi }}(z)}{J}_{m}(\frac{k\gamma \rho }{1+\frac{iz}{{z}_{R}}})\times {e}^{[-(\frac{1}{{w}^{2}(z)}-\frac{ik}{2{R}_{{\rm{g}}}(z)})\times ({\rho }^{2}+{\gamma }^{2}{z}^{2})]}{e}^{im\phi }$$

where *w(z)* is the radius of Bessel-Gaussian mode at distance *z* from its waist. *w*_0_ is the waist radius. The function *Φ(z)* = *k(1* − *γ*^2^*/2*)*z*-*arctan(z/z*_*R*_*)* describes the axial phase of the Bessel-Gaussian mode. *k* = 2*π/λ* is the wavenumber with *λ* the wavelength. *ρ* is the radial distance from the mode center. *γ* is the conicity angle of the Bessel beam. *z*_*R*_ is the Rayleigh range. *R*_*g*_ = *z* + *z*^2^_*R*_*/z* is the wavefront curvature of Bessel-Gaussian mode. *m* is the number of topological charge. φ is the azimuthal angle. To properly design the metasurface-based reflector, the phase and amplitude distributions of the lasing mode on two mirrors should be considered. The waist of the Bessel-Gaussian mode is set on mirror 1 (a plane mirror) and the radial phase on mirror 1 can be 0 or π. The mirror 2, having a distance of *L* away from the mirror 1, provides a phase-only reflection function written by

2 $$\phi (\rho )=-\,\frac{k{\rho }^{2}}{{R}_{g}(L)}\,-\,2\frac{k\gamma \rho }{1+\frac{{L}^{2}}{{{z}_{R}}^{2}}}$$

It is known that the phase function of a spherical mirror with a radius of *R* is −*kρ*^2^*/R* and that of an axicon is −*kαρ*. Hence, the phase function in Eq. (2) can be regarded as a combination of a spherical mirror and an axicon with properly selected *R* and *α*. Travelling twice through the axicon and replacing *z*_*R*_ with *kw*^2^_0_*/2*, *R* and *α* can be expressed as:

3 $$\alpha =\frac{\gamma }{1+4{L}^{2}/{(k{w}_{0}^{2})}^{2}}$$

4 $$R=L+\frac{{(k{w}_{0}^{2})}^{2}}{4L}$$

**Figure 3:**
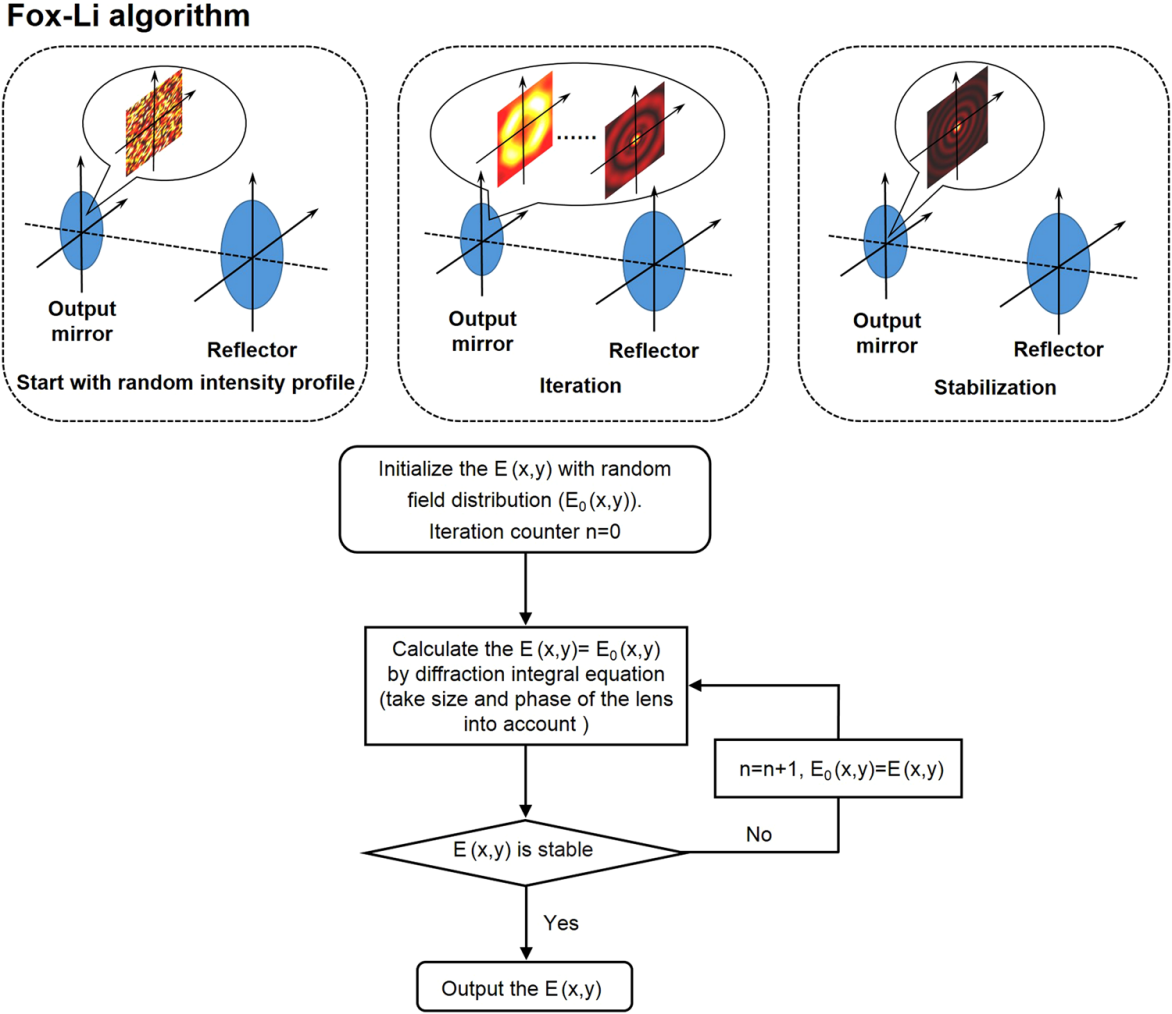
Principle and theory of Fox-Li algorithm.

Eqs (3, 4) give the mirror radius *R* and the axicon parameter *α* required to enable the lasing of Bessel-Gaussian mode with conicity angle of *γ* and waist radius of *w*_0_. Using Eqs (2–4) one can obtain the desired phase function for metasurface design. In addition, Bessel-Gaussian mode has properties of suppressed diffraction divergence and self-reconstruction. In the designed metasurface-assisted OAM-carrying Bessel-Gaussian laser, when maximizing the gain in the cavity volume, the maximum propagation distance of a Bessel-Gaussian mode *Z*_max_ is equal to the length of the cavity *L*, i.e. *Z*_max_ = *L* = *R*/(2tan*γ*), where *R* is the radius of the metasurface-based reflector. For the sake of manifesting the physical process of mode lasing and propagation in the laser resonator, Fox-Li algorithm^50^ (see Methods) can be applied to analyze the evolution of transverse electrical field, as shown in Fig. 3. To evaluate the quality of the generated Bessel-Gaussian modes, the mode purity or fidelity is introduced and defined as follows

5 $$p=\frac{{|\int {A}_{0}^{\ast }(x,y,z){A}_{t}(x,y,z)dxdydz|}^{2}}{\int {|{A}_{o}(x,y,z)|}^{2}dxdydz\int {|{A}_{t}(x,y,z)|}^{2}dxdydz}$$

where A_o_(x, y, z) and A_t_(x, y, z) are the complex amplitudes of the obtained and target Bessel-Gaussian mode, respectively.

## Simulations

**Figure 4:**
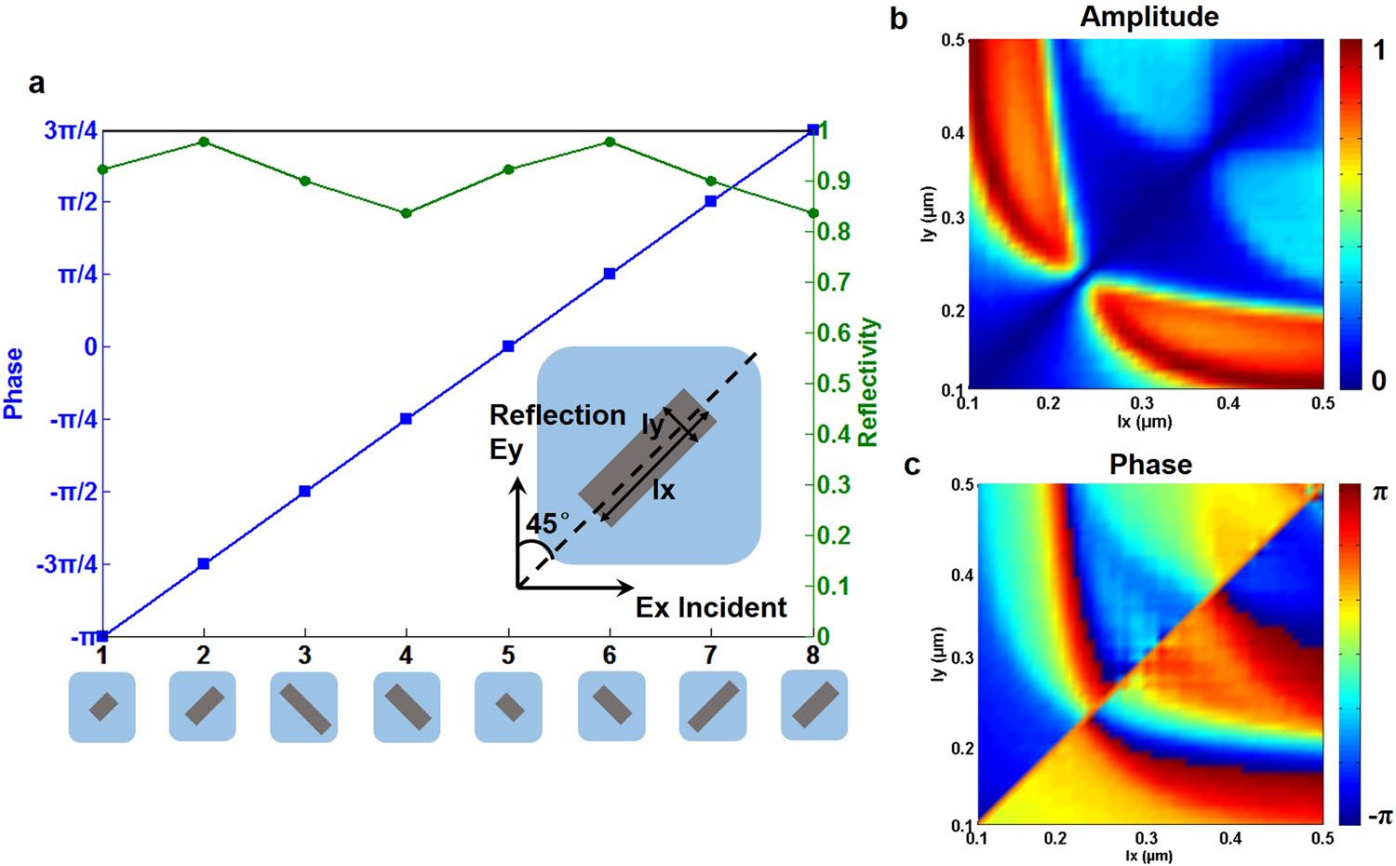
Characterization (phase and amplitude responses) of metasurface units. (**a**) Calculated phase shift and reflectivity of eight selected metasurface units with different geometric parameters. (**b**) Calculated amplitude distribution versus geometric parameters. (**c**) Calculated phase distribution versus geometric parameters.

According to the above principles and theories of metasurface-assisted OAM-carrying Bessel-Gaussian laser, we design and simulate the metasurface reflector. The cross-polarized scattering light including both amplitude and phase distributions for the metasurface unit are obtained with full-wave simulations using the finite difference time domain (FDTD) method^55^. The insets of Figs 1 and 4a illustrate the schematic of metasurface (structured Si antenna array) on SiO_2_ substrate with an Ag bottom layer. In the simulations, the thickness of Si, SiO_2_ and Ag is set to 250 nm, 200 nm and 150 nm, respectively. The size of the calculated square unit in the inset of Fig. 4a is 700 nm. The incident light is x-polarized, which can be decomposed into two perpendicular components correspond to the long and short axis of the unit rectangular Si resonator, respectively. The reflection amplitudes in both components are close to each other while the relative phase retardation is around π where the linear polarization conversion occurs, leading to a y-polarized reflected light. The dashed line in the inset of Fig. 4a indicates the symmetry axis of the metasurface unit. The angle between the polarization of incident light and symmetry axis is 45°. In addition, the gain medium is Nd:YVO_4_ and the lasing wavelength is 1064 nm. For a typical metasurface unit shown in the inset of Fig. 4a, by varying the geometric parameters of the rectangular unit, i.e. length l_x_ and width l_y_, the amplitude and phase distributions of the cross-polarized scattered light are calculated and plotted in Fig. 4b,c, respectively. One can clearly see from Fig. 4b,c that the normalized amplitude changes from 0 to 1 and the phase shift varies from −π to π. We choose eight kinds of units with different geometric dimensions as shown in Fig. 4a, providing a full phase coverage of 2π and a nearly constant amplitude.

**Figure 5:**
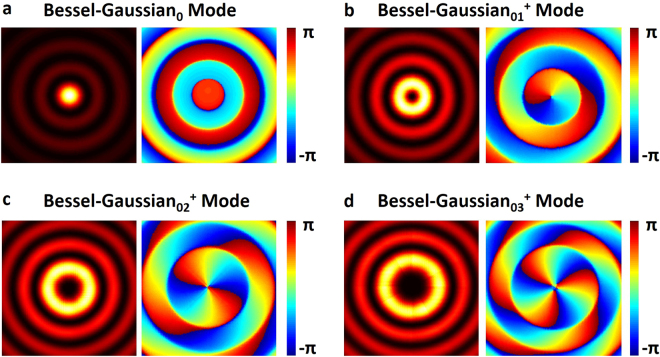
Ideal multi-ring intensity distribution and helical phase distribution of OAM-carrying Bessel-Gaussian modes without considering the real metasurface structure (perfect phase distribution in the right inset of Fig. 1 is used in the simulations). (**a**) Bessel-Gaussian_0_ mode. (**b**) Bessel-Gaussian_01_^+^ mode. (**c**) Bessel-Gaussian_02_^+^ mode. (**d**) Bessel-Gaussian_03_^+^ mode.

The employed metasurface reflector plays combined roles of an axicon and a spherical mirror. Using proper arrangement of eight selected units in Fig. 4a with a full phase coverage of 2π to construct a metasurface reflector can approximately provide the desired phase function in Eq. (2) for the lasing of Bessel-Gaussian modes. When we use the perfect phase distribution in the right inset of Fig. 1 in the simulations regardless of the real metasurface structure, the obtained results in Fig. 5 show almost ideal multi-ring intensity distribution and helical phase distribution of OAM-carrying Bessel-Gaussian modes.

**Figure 6:**
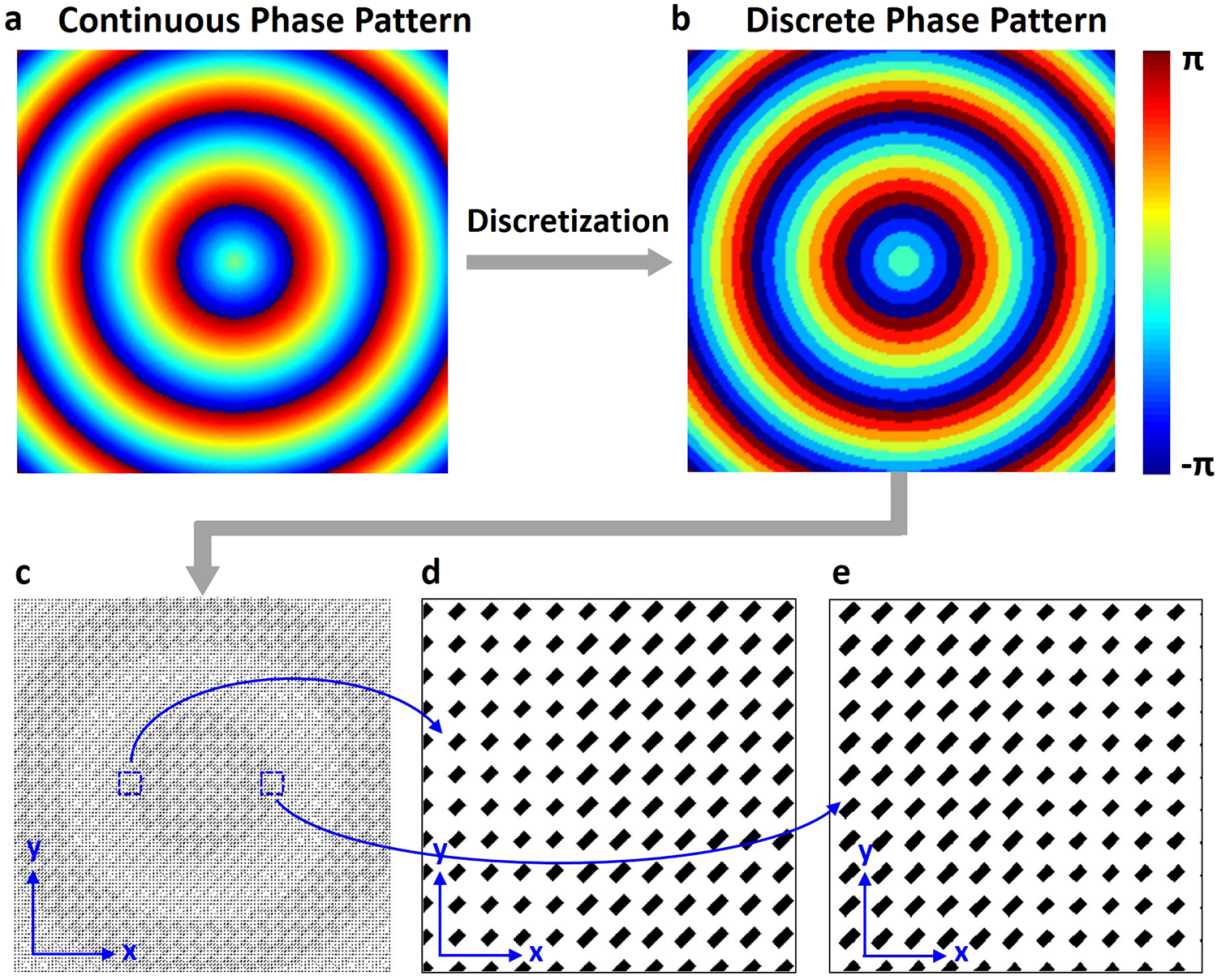
Discretization of continuous phase pattern and layout of metasurface structure. (**a**) Continuous phase pattern. (**b**) Discrete phase pattern. (**c**) Layout of metasurface structure corresponding to discrete phase pattern. (**d**,**e**) Zoom-in regions of the metasurface structure.

The desired perfect phase distribution of mirror 2 (combined functions of an axicon and a spherical mirror) in the right inset of Fig. 1 shows smooth phase change (i.e. continuous phase pattern). However, such continuous phase pattern is actually not achievable using metasurface structure which can be regarded as multiple pixels with discrete phase modulations. In view of the discrete characteristic of metasurface structure, it is necessary to replace the desired continuous phase pattern with a discrete one. In the simulations, we first discretize the continuous phase pattern. Figure 6b depicts the discrete phase pattern which discretizes the continuous phase pattern shown in Fig. 6a into eight values. Note that the discrete eight values are linked to eight selected metasurface units which provide a full phase coverage of 2π and a nearly constant amplitude. After the discretization, we then replace the discrete phase pattern by corresponding metasurface units, as shown in Fig. 6c–e. As a consequence, the designed metasurface structure is formed to enable the metasurface-assisted OAM carrying Bessel-Gaussian laser.

**Figure 7:**
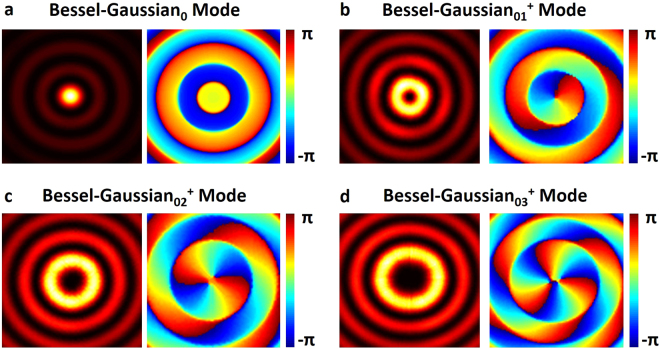
Simulated multi-ring intensity distribution and helical phase distribution of OAM-carrying Bessel-Gaussian modes in the designed metasurface-assisted Bessel-Gaussian laser (real metasurface structure and discontinuities of phase and amplitude responses are considered in the simulations). (**a**) Bessel-Gaussian_0_ mode. (**b**) Bessel-Gaussian_01_^+^ mode. (**c**) Bessel-Gaussian_02_^+^ mode. (**d**) Bessel-Gaussian_03_^+^ mode.

**Figure 8:**
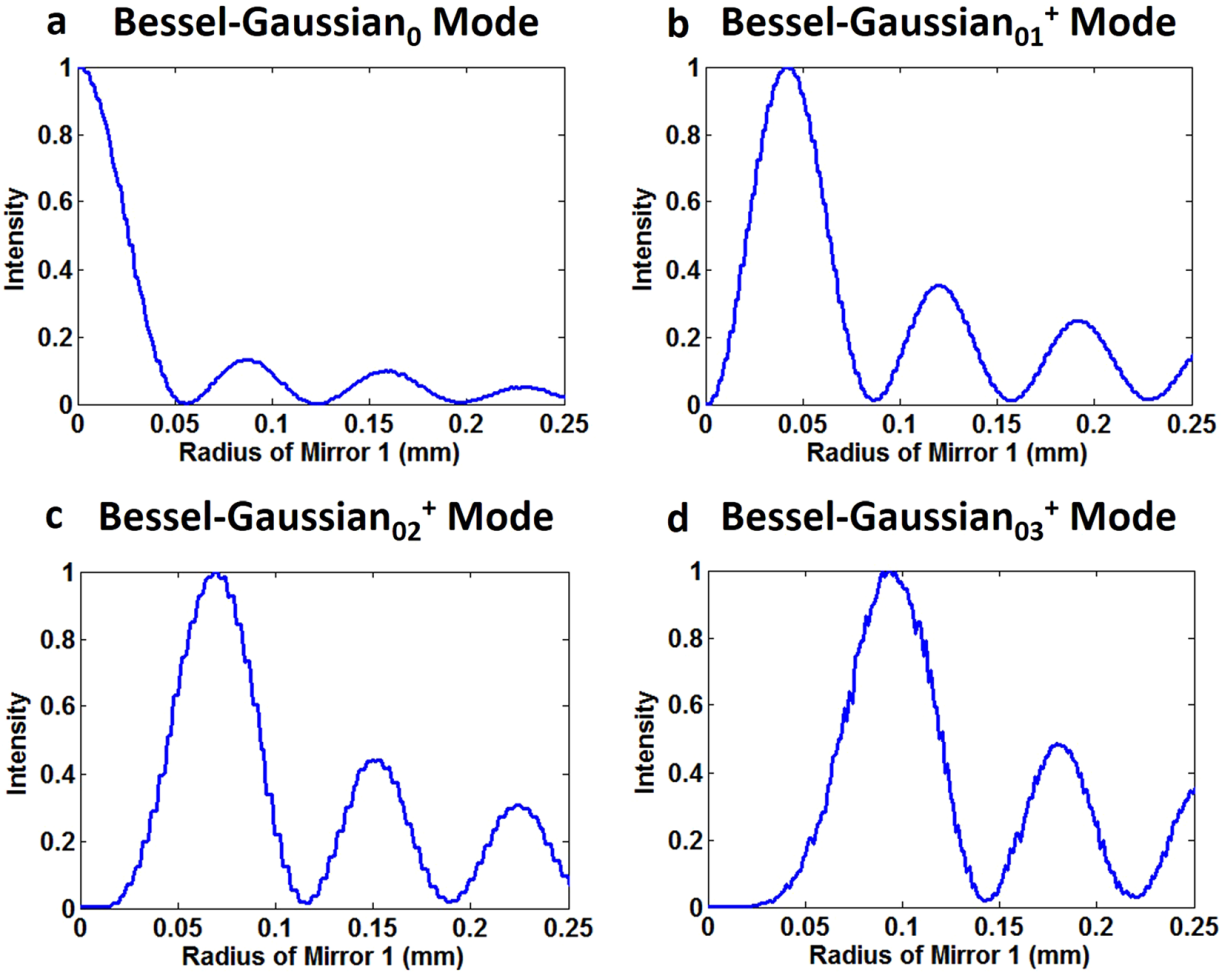
Simulated intensity profiles along the radial direction of Bessel-Gaussian modes corresponding to Fig. 7. (**a**) Bessel-Gaussian_0_ mode. (**b**) Bessel-Gaussian_01_^+^ mode. (**c**) Bessel-Gaussian_02_^+^ mode. (**d**) Bessel-Gaussian_03_^+^ mode.

Following the discretization process, the real metasurface structure and discontinuities in phase and amplitude responses are then taken into consideration in the simulations of laser with the Fox-Li algorithm. The output mirror in the laser influences the oscillation of Bessel-Gaussian modes of different order. It can be briefly explained with the fact that high-order Bessel-Gaussian modes suffer from relatively high loss. High-order Bessel-Gaussian modes might not be excited with limited size of the output mirror. To generate desired Bessel-Gaussian modes with different order, we adjust the radius of the output mirror as well as the MSE. Figure 7 shows simulation results with real metasurface structure. In the simulations, w_0_ of generated Bessel-Gaussian_0_, Bessel-Gaussian_+1_, Bessel-Gaussian_+2_ and Bessel-Gaussian_+3_ is 0.14 mm. γ of four Bessel-Gaussian modes is 0.6°. To generate Bessel-Gaussian modes with different orders, we appropriately adjust the radius of mirror 1. When the radius of mirror 1 is chosen to be 2.5 × w_0_, 2.6 × w_0_, 2.7 × w_0_, 2.8 × w_0_ and the radius of mirror 2 (metasurface-based reflector) is equal to 2Ltanγ, the Bessel-Gaussian_0_ to Bessel-Gaussian_+3_ modes can be excited, respectively. When carefully comparing the real metasurface structure results in Fig. 7 to those ideal results in Fig. 5, one can see slight performance degradation (circular symmetry) of the laser when considering the real metasurface structure and the discontinuities in phase and amplitude responses. However, the obtained multi-ring intensity distribution and helical phase distribution still show the successful implementation of metasurface-assisted OAM-carrying Bessel-Gaussian laser with favorable operation performance (high laser beam quality). The lasing Bessel-Gaussian_0_, Bessel-Gaussian_01_^+^, Bessel-Gaussian_02_^+^ and Bessel-Gaussian_03_^+^ modes show high fidelities of ~0.889, ~0.889, ~0.881 and ~0.879, respectively. Figure 8 plots the simulated intensity profiles along the radial direction of Bessel-Gaussian modes corresponding to Fig. 7.

**Figure 9:**
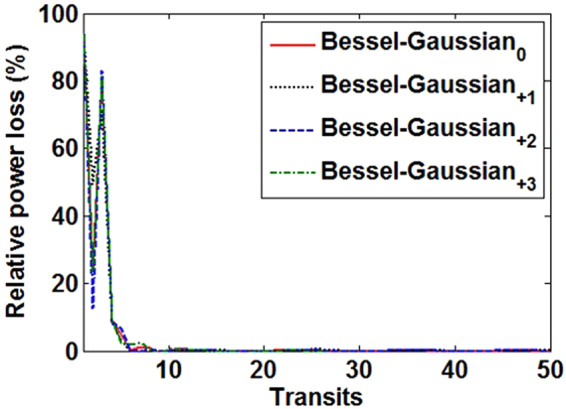
Relative power loss versus transits for different Bessel-Gaussian modes (m = 0, +1, +2, +3).

To assess the convergence and stability of the Fox-Li algorithm, we analyze the behavior of the relative power loss per round trip, *δ*_*k*_ = (*I*^*k*^ − *I*^*k*+1^)/*I*^*k*^, where *I*^*k*^ = ∫|*u*(*x*, *y*)|^2^dxdy is the integral of the field intensity at the *k*th transit. The behavior of the relative power loss as a function of the transits is shown in Fig. 9. We observe that there is a transient state around which the mode oscillates with high loss. Such transient state is unstable. However, after that the system becomes stable with low loss. During the transient process, the field profile evolves to the Bessel-Gaussian mode profile with m = 0, 1, 2 or 3. Once the Bessel-Gaussian mode is stabilized, the low loss remains constant.

**Figure 10:**
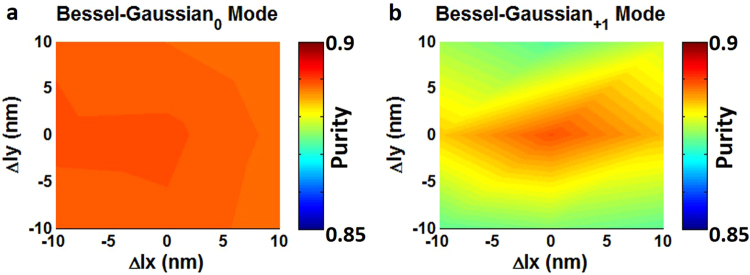
Simulated Bessel-Gaussian mode purity versus fabrication errors of metasurface structure. (**a**) Bessel-Gaussian_0_ mode. (**b**) Bessel-Gaussian_01_^+^ mode.

We study the fabrication tolerance of the metasurface-assisted OAM-carrying Bessel-Gaussian laser. Figure 10 shows simulated Bessel-Gaussian mode purity as a function of the fabrication errors (Δl_x_, Δl_y_) of metasurface structure, where Δl_x_ and Δl_y_ are the geometric offset of the fabricated eight metasurface units from the designed ones. We choose Bessel-Gaussian_0_ mode and Bessel-Gaussian_01_^+^ mode as typical examples. As shown in Fig. 10a, for Bessel-Gaussian_0_ mode the maximum mode purity is ~0.889 (Δl_x_ = 0, Δl_y_ = 0). With fabrication errors varying from −10 to 10 nm, the mode purity of Bessel-Gaussian_0_ mode is larger than 0.887. For Bessel-Gaussian_+1_ mode the maximum mode purity is also ~0.889 (Δl_x_ = 0, Δl_y_ = 0), as shown in Fig. 10b. When the fabrication errors vary from −10 to 10 nm, the mode purity of Bessel-Gaussian_+1_ mode is larger than 0.873. The obtained results indicate that the designed metasurface-assisted OAM-carrying Bessel-Gaussian laser shows favorable tolerance to the fabrication errors. Additionally, high-order Bessel-Gaussian modes show increased sensitivity to the fabrication errors compared to low-order Bessel-Gaussian modes such as Bessel-Gaussian_0_ mode.

**Figure 11:**
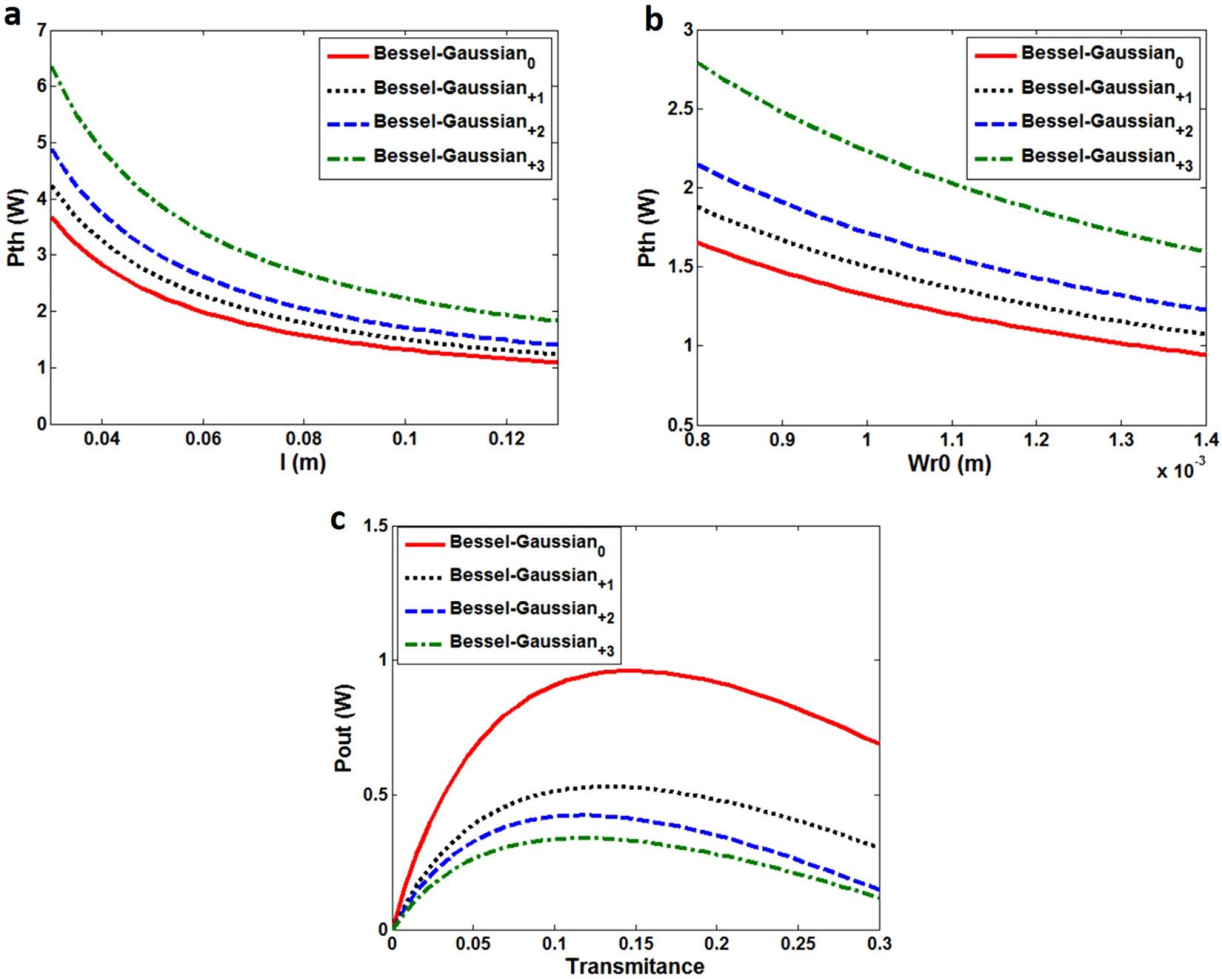
Characterization of the lasing properties of the metasurface-assisted OAM-carrying Bessel-Gaussian laser. (**a**) Simulated threshold power (Pth) versus length of gain medium (l). Wr0 = 0.8 mm. (**b**) Simulated threshold power (Pth) versus beam radius of pump (Wr0). L = 0.1 m. (**c**) Simulated output lasing power versus transmittance of mirror 1.

We further characterize the lasing properties of the metasurface-assisted OAM-carrying Bessel-Gaussian laser. Figure 11a,b show the simulated threshold power (Pth) as functions of the length of gain medium (*l*) and beam radius of pump (Wr0). One can clearly see that the threshold power decreases with the increase of the length of gain medium and beam radius of pump. Also, higher-order Bessel-Gaussian modes show relatively higher threshold power. Figure 11c describes the relationship between the output lasing power and the transmittance of mirror 1. It is shown that there exists an optimal transmittance of mirror 1 giving a maximum output lasing power. The maximum output power is around 0.91 W under 5 W pump for the zero-order Bessel-Gaussian mode (Bessel-Gaussian_0_). In addition, the output lasing power decreases for high-order Bessel-Gaussian modes.

The obtained results shown in Figs 4–11 indicate the successful implementation of the metasurface-assisted OAM-carrying Bessel-Gaussian laser with favorable operation performance.

## Discussion

In summary, we design an OAM-carrying Bessel-Gaussian laser structure consisting of a FP cavity, a pump, a gain medium, and an MSE. The FP cavity is formed by two mirrors, i.e. one partially transparent output plane mirror and the other metasurface-based reflector mirror. The MSE benefits the direct generation of Bessel-Gaussian modes with controllable handedness of the helical phase. The dielectric metasurface with compact structure, providing flexible spatial light manipulation, replaces the traditional complicated volume axicon and spherical mirror. Zero-order and high-order Bessel-Gaussian lasing modes are achievable by properly changing the size of output mirror and putting MSE in the cavity. We also simulate and characterize the designed metasurface-assisted OAM-carrying Bessel-Gaussian laser using the Fox-Li algorithm, manifesting the physical process of mode lasing and propagation in the cavity. The simulation results show successful lasing of Bessel-Gaussian_0_, Bessel-Gaussian_01_^+^, Bessel-Gaussian_02_^+^ and Bessel-Gaussian_03_^+^ modes with high fidelities of ~0.889, ~0.889, ~0.881 and ~0.879, respectively. The convergence and stability of the Fox-Li algorithm and the fabrication tolerance are studied. We also discuss the threshold power and output lasing power as functions of the length of gain medium, beam radius of pump and transmittance of output mirror. The obtained results show favourable operation performance.

Bessel-Gaussian mode with OAM order of 3 is achieved in the simulations. Remarkably, the order of generated Bessel-Gaussian mode is determined by several factors. The structure of laser resonator, the loss and pump efficiency of high-order Bessel-Gaussian modes are the main determining factors. The structure of laser resonator is affected by the resonance cavity length, radius of curvature, dimensions of reflector (mirror 2) and radius of output mirror 1. The loss of high-order modes includes diffraction loss influenced by the conicity angle of the Bessel beam and structure loss induced by axial nonuniformity and fabrication errors of metasurface-assisted reflector (mirror 2). For pump efficiency, it is affected by the length of the gain medium and beam radius of pump. In the designed metasurface-assisted OAM-carrying Bessel-Gaussian laser, the conventional axicon together with the spherical mirror is replaced by the metasurface-based reflector. The simulation results show that the real metasurface structure and its induced discontinuities in phase and amplitude responses have slight impact on the performance degradation of the generated OAM-carrying Bessel-Gaussian modes. All these factors could have influence on the order of generated Bessel-Gaussian modes.

The presented metasurface-assisted OAM-carrying Bessel-Gaussian laser may further find interesting applications not only in optical communication but also in non-communication fields.

## Methods

Fox-Li algorithm is used to manifest the physical process of mode lasing and propagation in the laser resonator. Firstly, the phase and amplitude of laser beams start with random distribution, i.e. initializing the electrical field with random field distribution. Then, taking the diffraction integral equation method for simulations. The beams travel twice through the resonator and are reflected once by the metasurface-based reflector. After that, comparing the calculated results with the previous transverse electrical field to find whether the field distribution profile of beams tends to be stable. If not, then repeating the iterative process until it becomes stable and the desired Bessel-Gaussian mode will be established.
